# Identification of a novel miRNA-based recurrence and prognosis prediction biomarker for hepatocellular carcinoma

**DOI:** 10.1186/s12859-022-05040-y

**Published:** 2022-11-14

**Authors:** Xuanfeng Zhang, Dong Zhang, Xuefeng Bu, Xinhui Zhang, Long Cui

**Affiliations:** 1 Center of Hepatobiliary Pancreatic Disease, XuZhou Central Hospital, Xuzhou, Jiangsu People’s Republic of China; 2 Bengbu Medical College, Bengbu, Anhui People’s Republic of China; 3 Department of General Surgery, Affiliated People’s Hospital of Jiangsu University, Zhenjiang, Jiangsu People’s Republic of China

**Keywords:** Hepatocellular Carcinoma, Recurrence, Prognosis

## Abstract

**Background:**

A high recurrence rate has always been a serious problem for treatment of hepatocellular carcinoma (HCC). Exploring predictors of postoperative and posttransplantation recurrence in patients with HCC can guide treatment strategies for clinicians.

**Results:**

In this study, logistic regression and multivariate Cox regression models were constructed with microRNA expression profile data from The Cancer Genome Atlas (TCGA) and gene expression omnibus (GEO). The accuracy of predictions was assessed using receiver operating characteristic curve (ROC) and Kaplan‒Meier survival curve analyses. The results showed that the combination of 10 miRNAs (including hsa-miR-509-3p, hsa-miR-769-3p, hsa-miR-671-3p, hsa-miR-296-5p, hsa-miR-767-5p, hsa-miR-421, hsa-miR-193a-3p, hsa-miR-139-3p, hsa-miR-342-3p, and hsa-miR-193a-5p) accurately predicted postoperative and posttransplantation malignancy recurrence in HCC patients and was also valuable for prognostic evaluation of HCC patients. The 10-miRNA prediction model might assist doctors in making prognoses for HCC patients who have a high probability of relapse following surgery and in offering additional, individualized treatment to lessen that risk.

## Introduction

Hepatocellular carcinoma (HCC) is the sixth most common and the third deadliest malignant tumor globally [1]. The tumorigenic process of HCC is related to hepatitis virus infection, alcoholic liver cirrhosis, and nonalcoholic fatty liver disease [2]. Surgical resection, which can significantly prolong the survival time of patients, is still the best choice for patients with early or advanced HCC [3]. In addition, liver transplantation (LT) is considered to be an option for treatment of liver cancer. Because LT has the ability to cure liver disease, it is a better choice in certain circumstances (e.g.. small hepatocellular r carcinoma) [4]. Radiotherapy, radiofrequency ablation (RFA), and transcatheter arterial chemoembolization (TACE) are options for patients with liver cancer who cannot undergo resection and liver transplantation [5]. All these treatments have significantly improved the prognosis of patients with HCC.

Despite extension of the overall survival of HCC, postoperative recurrence still has a serious impact on these patients. Postoperative recurrence of HCC mainly includes two types, intrahepatic metastasis (IM) and multicenter occurrence (MO) [6]. The tumor recurrence rate at 5 years after liver resection can reach 70% [7]. However, patients face the same problem after liver transplantation, especially recipients whose tumor burden exceeds liver transplantation criteria [8]. Patients who experience relapse have worse prognosis and shorter survival, with a median survival of approximately 9 to 16 months [9]. Finding predictors of HCC recurrence can guide clinicians in the choice of treatment for patients postresection and posttransplantation.

However, most of the current research focuses on the prediction of the prognosis of patients with HCC, and the number of studies related to the prediction of liver cancer recurrence is relatively small. Some researchers have been paying attention to the assessment of recurrence of HCC patients, and have used mRNAs to build a prediction model of recurrence of HCC patients [10]. We believed that miRNAs have unique advantages in humoral assays compared to mRNAs. Some researchers have the same idea as us. They constructed a miRNA-based liver cancer recurrence prediction model. The variable screening method used was univariate Cox analysis, and the modeling method was multivariate Cox analysis [11]. On this basis, we designed an innovative variable screening method (not just the common Cox regression analysis and Lasso regression analysis) to establish a Logistic classification-based model for predicting HCC recurrence in postresection and posttransplantation HCC patients. Internal and external validations showed that this is a stable method for predicting tumor recurrence in HCC patients.

## Method

### Data collection and processing

RNA-seq data of miRNA mature strand expression (Cancer Genome Atlas liver cancer, LIHC) were downloaded from UCSC xena (http://xena.ucsc.edu/) and normalized to log(RPM + 1). ChIP data (GSE30297) of miRNA mature strand expression were downloaded from GEO (https://www.ncbi.nlm.nih.gov/geo/). LIHC was used for training and internal validation and GSE30297 for recurrence external validation. To avoid bias in calculating risk scores, we removed miRNAs expressed at relatively low levels in all samples. This data processing was performed using R software (version 4.2.0).

### Training set splitting and variable selection

The LIHC cohort was divided into a training dataset (60%) and an internal validation dataset (40%). The LIHC training dataset was used for recurrence and survival prediction model construction. Collinearity was removed using LASSO regression analysis. We reset the lambda value in the LASSO program and reduced the number of miRNAs included in the analysis to 10. This process was carried out in R software, and the survival (version 3.1.1) and glmnet (version 4.1.4) R packages were used.

### Gaussian mixture model for further variable screening

All chosen miRNAs were merged, permuted, and modeled using logistic regression analysis. The AUC was computed individually for each model. The classification used model-based hierarchical agglomerative clustering, which is based on a Gaussian finite mixture model, and the AUC value was used as the classification basis. The mclust R package (version 5.4.9) was utilized for model-based clustering, classification, and density estimation based on finite normal mixture modeling.

### Predictive model generation

Recurrence prediction models were established using logistic regression, and prognostic models were established using multivariate Cox regression. Model building was performed with the LIHC training dataset.

### Data visualization

ROC curves were used to demonstrate model discrimination, and Kaplan‒Meier curves were used to demonstrate the relationship between miRNA expression and overall or disease-free survival. The miRNA expression heatmap was displayed using the pheatmap package (version 1.0.12). Functional analysis was performed using clusterProfiler (version 4.4.2), and the results were visualized using gglopt2 (version 3.3.5).

### Statistical analysis

R software was used for all statistical analyses (version 4.2.0). Logistic regression was utilized to predict tumor recurrence. Cox regression and Kaplan‒Meier analysis were employed in the survival study. We performed t tests for paired samples. A p value of 0.05 or less was used to determine significance in all statistical tests.

## Result

### Variable filtering

**Fig. 1:**
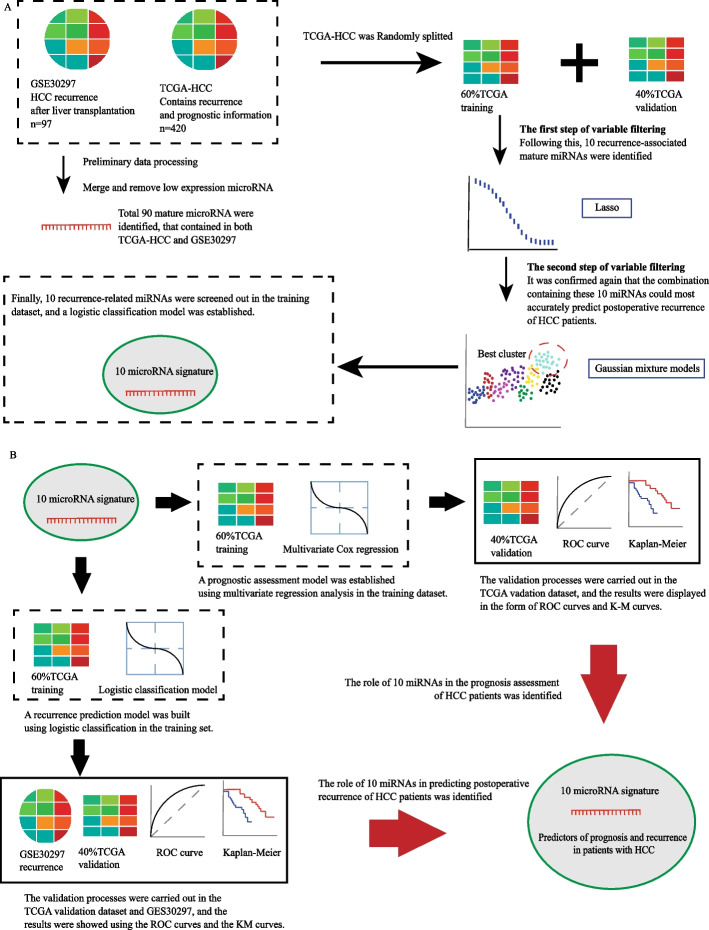
Workflow of this study

**Fig. 2:**
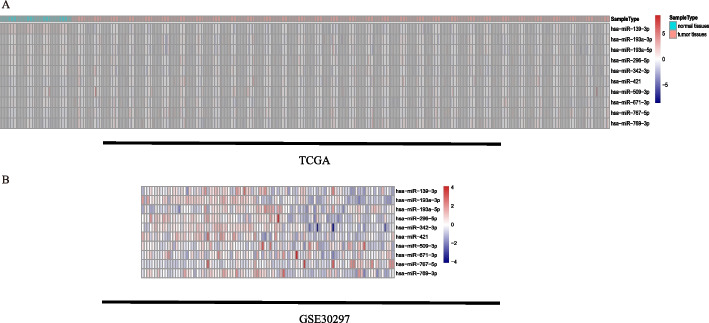
The miRNA expression heatmap. **A** Expression levels of 10 miRNAs in TCGA. **B** Expression levels of 10 miRNAs in GSE30297. Explanatory note: Among these 10 miRNAs, expression of hsa-miR-767-5p was significantly upregulated in HCC tissues and that of hsa-miR-139-3p was significantly downregulated in HCC tissue; differences in expression of the other miRNAs were not statistically significant

Previous studies have tended to use differentially expressed genes to screen for molecules that may be associated with worse prognosis, such as recurrence, in HCC patients. We believe that prognostic or recurrence-related genes should be differentially expressed in different risk groups of tumors and not in tumors or normal tissues. Therefore, we included all mature miRNAs in the first step of variable screening. A total of 90 miRNAs were obtained after taking the intersection of LIHC, GSE30297, and GSE31384 data and deleting miRNAs with low expression (Fig. 1A). The characteristics of the three datasets (e.g., sample size, platform) are shown in Table 1. As depicted in Fig. 1A, after we reset the lambda parameters in LASSO regression, a total of 10 miRNAs were identified for further analysis (hsa-miR-509-3p, hsa-miR-769-3p, hsa-miR-671-3p, hsa-miR-296-5p, hsa-miR-767-5p, hsa-miR-421, hsa-miR-193a-3p, hsa-miR-139-3p, hsa-miR-342-3p, and hsa-miR-193a-5p). The expression levels of these 10 candidate miRNAs in the three datasets are shown in Fig. 2.

**Table 1:** Characteristics of the datasets involved in this study

Dataset	Number of samples	Clinical features	Application	Platform
miRNA mature strand expression				
TCGA liver cancer (LIHC)	420	Recurrence and survival	Training and validation	RNA-seq
GSE30297	97	Recurrence	Validation	GPL8786

### Gaussian mixture model for further variable screening

**Fig. 3:**
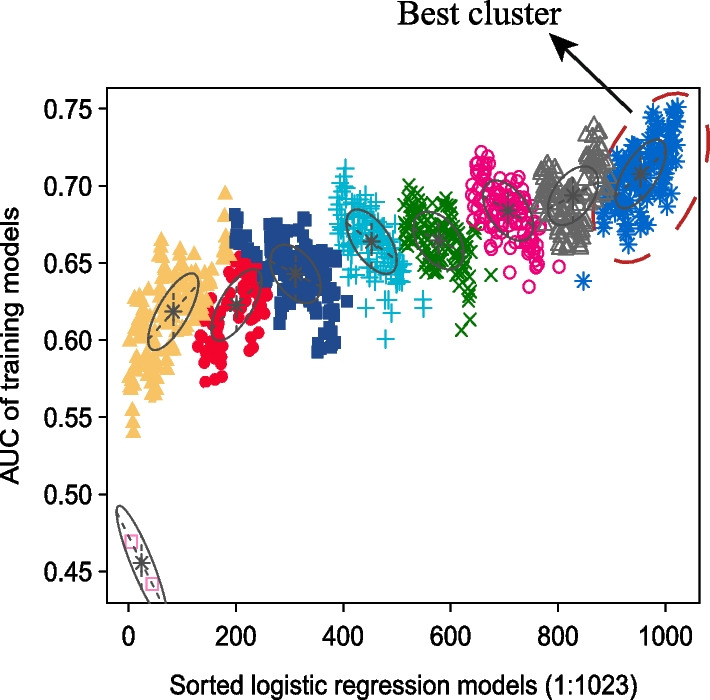
The diagram of the Gaussian mixture model cluster analysis result. The cluster with the highest AUC values is marked with dashed lines in the figure

We performed classification using a Gaussian finite mixture model to further fit the model and more precisely estimate recurrence (Fig. 1A). A total of 1023 logistic regression models were produced after the permutation and combination of 10 miRNAs. All logistic regression models were divided into 9 clusters. Among them, the cluster in the upper right corner of Fig. 3 had the highest AUC value. The NO.1023 model, i.e., the model incorporating all 10 miRNAs, had the largest AUC value (AUC = 0.75). The Gaussian mixture model further determined that among all models, that incorporating all 10 miRNAs had the best predictive capability.

### Logistic regression for recurrence prediction

**Fig. 4:**
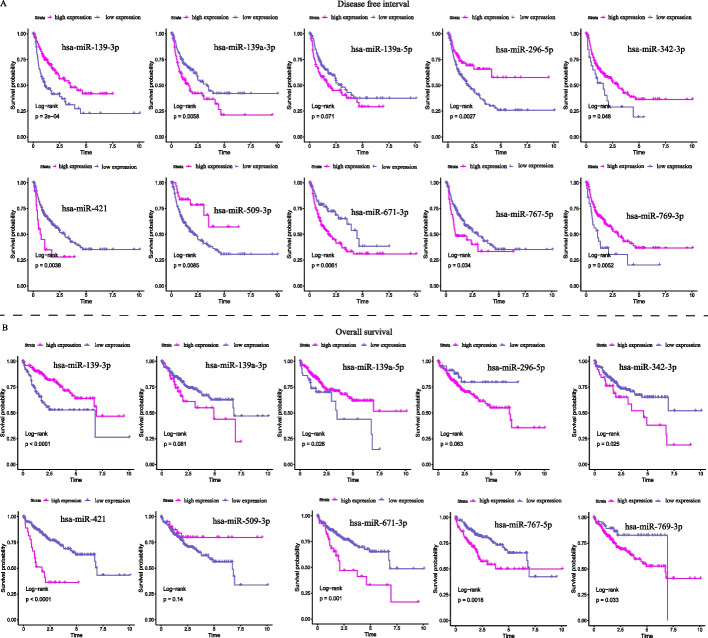
Kaplan‒Meier survival analysis estimates of the DFI and OS of HCC patients according to expression of the 10 miRNAs. **A** Kaplan‒Meier analysis of the disease-free interval (DFI). **B** Kaplan‒Meier analysis of overall survival (OS)

The LIHC cohort was randomly divided into 2 groups (60% TCGA training set and 40% TCGA validation set). The logistic regression model development process used the 60% TCGA training set. The results showed that the combination of hsa-miR-509-3p (coef = − 0.378, *P* = 0.09), hsa-miR-769-3p (coef = − 0.857, *P* = 0.01), hsa-miR-671-3p (coef = 0.881, *P* = 0.009), hsa-miR-296-5p (coef = − 0.219, *P* = 0.137), hsa-miR-767-5p (coef = − 0.128, *P* = 0.08), hsa-miR-421 (coef = − 0.152, *P* = 0.58), hsa-miR-193a-3p (coef = 0.412, *P* = 0.25), hsa-miR-139-3p (coef = − 0.356, *P* = 0.04), hsa-miR-342-3p (coef = 0.463, *P* = 0.02), and hsa-miR-193a-5p (coef = 0.412, *P* = 0.28) comprised a tumor recurrence predictor for HCC patients. Recurrence is associated with the disease-free interval (DFI) in malignancy. The relationship between the 10 miRNAs and DFI is demonstrated in Fig. 4A, where expression of hsa-miR-139-3p, hsa-miR-296-5p, hsa-miR-342-3p, hsa-miR-509-3p, and hsa-miR-769-3p correlated positively with DFI and that of hsa-miR-193a-3p, hsa-miR-421, hsa-miR-671-3p, and hsa-miR-767-5p correlated negatively.

**Fig. 5:**
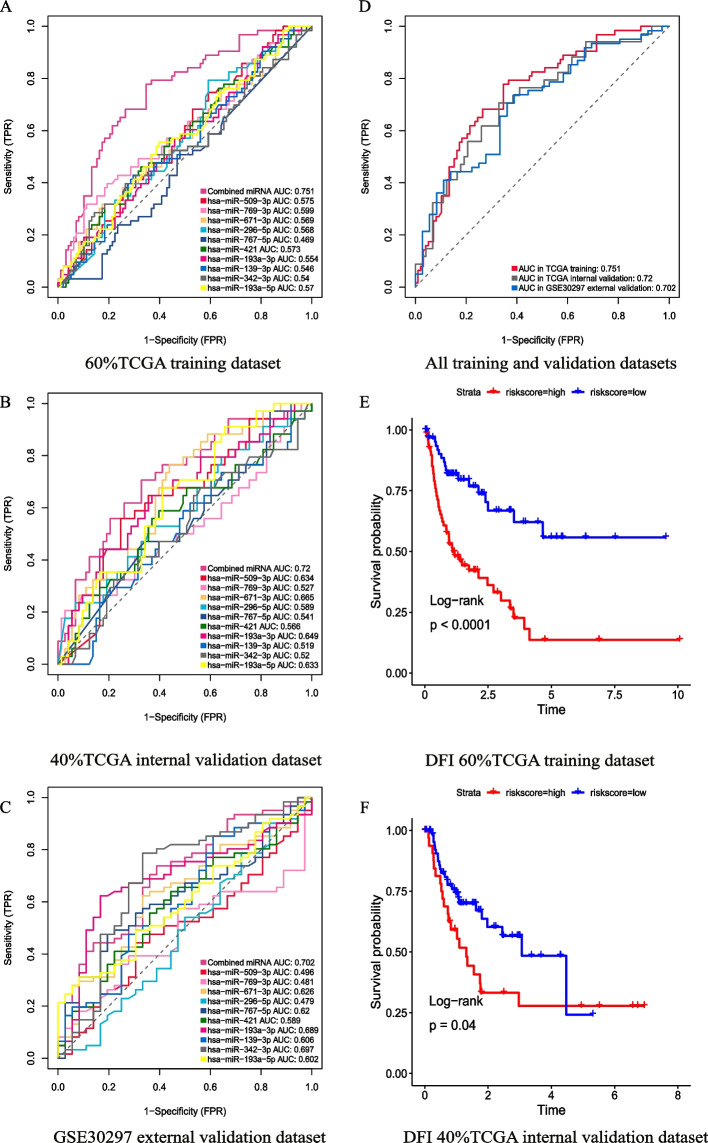
Predictive ability of the combination of 10 miRNAs for postoperative recurrence and posttransplantation recurrence in HCC patients. **A** ROC curves of the 10-miRNA combination and individual miRNAs in the training dataset from TCGA. **B** ROC curves of the 10-miRNA combination and individual miRNAs in the validation dataset from TCGA. **C** ROC curves of the 10-miRNA combination and individual miRNAs in the GSE30297 posttranslation recurrence validation dataset. **D** ROC curves of the 10-miRNA combination in the training and validation datasets. **E** Kaplan‒Meier analysis of the disease-free interval (DFI) in the training dataset from TCGA. **F** Kaplan‒Meier analysis of overall survival (OS) in the validation dataset from TCGA

To further validate the recurrence predictive capability of these 10 miRNAs, logistic regression risk scores were calculated using the following formula: the combination miRNA panel = (− 0.378 × expression of hsa-miR-509-3p) + (− 0.857 × expression of hsa-miR-769-3p) + (0.881 × expression of hsa-miR-671-3p) + (− 0.219 × expression of hsa-miR-296-5p) + (− 0.128 × expression of hsa-miR-767-5p) + (− 0.152 × expression of hsa-miR-421) + (0.412 × expression of hsa-miR-193a-3p) + (− 0.356 × expression of hsa-miR-139-3p) + (0.463 × expression of hsa-miR-342-3p) + (0.412 × expression of hsa-miR-193a-5p). The combination of 10 miRNAs in the training dataset from TCGA had the highest AUC value (AUC = 0.751) compared to individual miRNAs (hsa-miR-509-3p = 0.575, hsa-miR-769-3p = 0.599, hsa-miR-671-3p = 0.569, hsa-miR-296-5p = 0.568, hsa-miR-767-5p = 0.469, hsa-miR-421 = 0.573, hsa-miR-193a-3p = 0.554, hsa-miR-139-3p = 0.546, hsa-miR-342-3p = 0.54, hsa-miR-193a-5p = 0.57). This result showed that the combination of these 10 miRNAs had better capability to predict postoperative recurrence in HCC patients than the single miRNAs (Fig. 5A). Additionally, Kaplan‒Meier survival analysis was utilized to evaluate patient DFS between the high-risk group and the low-risk group, with the former having a much greater recurrence rate than the latter (Fig. 5E).

The same result was verified again in the internal validation dataset from TCGA, which showed that the combination of the 10 miRNAs had the best predictive ability for recurrence after surgery (AUC = 0.72) (Fig. 5B). The AUC values for hsa-miR-509-3p, hsa-miR-769-3p, hsa-miR-671-3p, hsa-miR-296-5p, hsa-miR-767-5p, hsa-miR-421, hsa-miR-193a-3p, hsa-miR-139-3p, hsa-miR-342-3p, and hsa-miR-193a-5p were 0.634, 0.527, 0.665, 0.589, 0.541, 0.566, 0.649, 0.519, 0.52, and 0.633, respectively (Fig. 5B). In the validation set, the combination of the 10 miRNAs still had the best postoperative recurrence prediction capability. As with the training dataset from TCGA, Kaplan‒Meier survival analysis was used to compare the difference in DFI between the high- and low-risk groups, and the results showed a higher recurrence rate in the former (Fig. 5F).

In addition to surgery, liver transplantation is an important treatment for HCC, though recurrence after liver transplantation is an important factor affecting the survival time of patients. To further validate the ability of the 10 miRNAs to predict HCC recurrence in patients after liver transplantation, we used GSE30297 as an external validation dataset. Surprisingly, these 10 miRNAs were also able to accurately predict liver cancer recurrence after liver transplantation (AUC = 0.702) (Fig. 5C). Again, the combination of the 10 miRNAs was able to predict recurrence after liver transplantation more accurately than any single miRNA (Fig. 5C).

In summary, the logistic regression formula constructed from 10 miRNAs was able to accurately predict tumor recurrence in postresection and posttransplantation HCC patients and performed well in TCGA training, TCGA internal validation, and GSE30297 external validation datasets (Fig. 5D).

### Multivariate Cox regression for overall survival prediction

The logistic regression model constructed with the 10 miRNAs was able to accurately predict tumor recurrence after liver resection and liver transplantation, and the risk score correlated with DFI in patients with HCC (Fig. 1B). To further validate the ability of the 10 miRNAs to predict survival in HCC patients, we constructed a multivariate Cox regression model (Fig. 1B).

Multivariate regression analysis showed that combination of hsa-miR-509-3p (HR: 1.085, 95% CI 0.777–1.516; *P* = 0.631), hsa-miR-769-3p (HR: 0.752, 95% CI 0.386–1.464; *P* = 0.402), hsa-miR-671-3p (HR: 2.613, 95% CI 1.381–4.942; *P* = 0.003), hsa-miR-296-5p (HR: 0.877, 95% CI 0.644–1.194; *P* = 0.403), hsa-miR-767-5p (HR: 1.03, 95% CI 0.911–1.165; *P* = 0.638), hsa-miR-421 (HR: 1.097, 95% CI 0.658–1.798; *P* = 0.744), hsa-miR-193a-3p (HR: 1.702, 95% CI 0.864–3.355; *P* = 0.124), hsa-miR-139-3p (HR: 0.677, 95% CI 0.478–0.959; *P* = 0.028), hsa-miR-342-3p (HR: 1.102, 95% CI 0.785–1.548; *P* = 0.574), and hsa-miR-193a-5p (HR: 0.583, 95% CI 0.28–1.215; *P* = 0.15) was a prognostic factor in HCC. Multivariate regression risk scores were calculated using the following formula: the combination miRNA panel = (0.082 × expression of hsa-miR-509-3p) + (− 0.285 × expression of hsa-miR-769-3p) + (0.96 × expression of hsa-miR-671-3p) + (− 0.132 × expression of hsa-miR-296-5p) + (0.03 × expression of hsa-miR-767-5p) + (0.0839 × expression of hsa-miR-421) + (0.532 × expression of hsa-miR-193a-3p) + (− 0.39 × expression of hsa-miR-139-3p) + (0.097 × expression of hsa-miR-342-3p) + (− 0.539 × expression of hsa-miR-193a-5p). Multivariate Cox regression analysis showed that miRNAs with HR values less than 1 were negatively associated with OS, which was different from the results of Kaplan‒Meier analysis for the individual miRNAs (Fig. 4B).

**Fig. 6:**
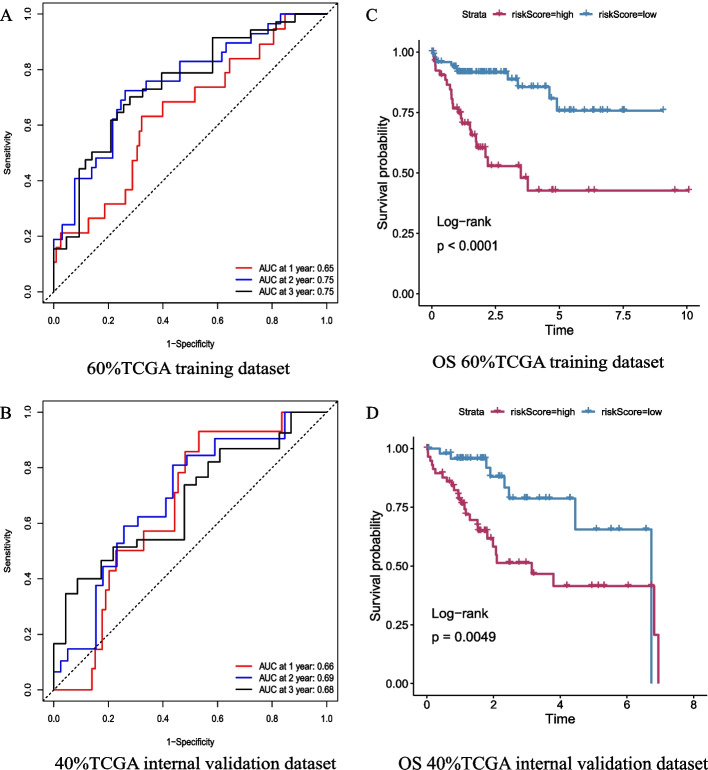
The role of the 10-miRNA combination in prognostic assessment of HCC patients. **A** ROC curves showed that the Cox regression model constructed using these 10 miRNAs in the training dataset from TCGA could well predict the prognosis of HCC patients. **B** ROC curves showed that this 10-miRNA combination also performed well in the validation dataset from TCGA. **C** Kaplan‒Meier survival analysis estimates of the OS of HCC patients according to risk scores calculated with this 10-miRNA combination in the training dataset. **D** Kaplan‒Meier survival analysis estimates of the OS of HCC patients according to the risk score calculated with this 10-miRNA combination in the validation dataset

Using the training dataset from TCGA, the combination of the 10 miRNAs was well able to predict the 3-year survival status of HCC patients (1 year = 0.65, 2 years = 0.75, and 3 years = 0.75) (Fig. 6A). The Kaplan‒Meier curve showed that patients in the high-risk subgroup had worse prognosis (Fig. 6C).

The same conclusion was confirmed in the internal validation dataset from TCGA. The ROC curve showed the combination of the 10 miRNAs to be a desirable predictor of overall survival outcome (1 year = 0.66, 2 years = 0.69, and 3 years = 0.68) (Fig. 6B). Patients were divided into high-risk and low-risk groups according to risk score, and survival analysis suggested that the high-risk group had a significantly higher death rate than the low-risk group (Fig. 6D).

In conclusion, these 10 miRNAs were also good predictors of overall survival in patients with HCC. A multivariate Cox regression model constructed with these 10 miRNAs to calculate risk score was able to assess the prognosis of HCC patients.

Identification of the functions of the 10 miRNAs.

**Fig. 7:**
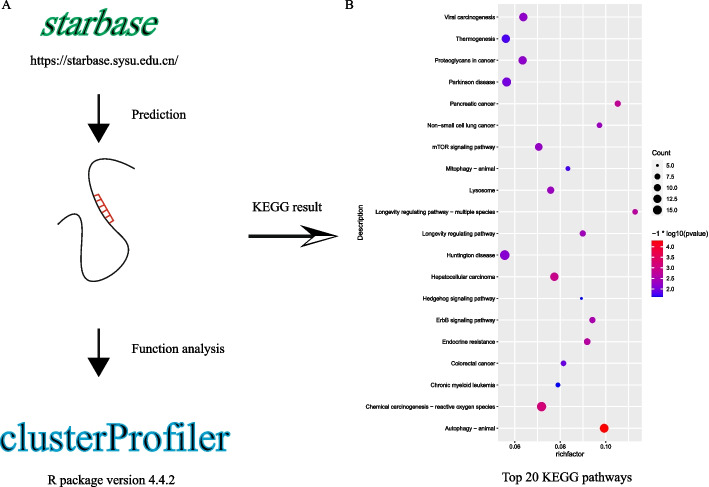
Results of functional analysis of 10 miRNAs. **A** Flowchart of functional analysis. **B** Bubble chart showing the top 20 pathways related to the 10 miRNAs

Because these 10 miRNAs are associated with the prognosis of liver cancer patients, exploring their functions is crucial for subsequent studies. Binding partners for these 10 miRNAs were predicted using the StarBase database, and then clusterProfiler was used for Kyoto Encyclopedia of Genes and Genomes (KEGG) pathway analysis (Fig. 7). Functional analysis showed the 10 miRNAs to be enriched in autophagy, hepatocellular carcinoma, the ErbB signaling pathway, and the mTOR signaling pathway, as shown in the bubble chart in Fig. 7.

This result suggests that these 10 miRNAs might influence the biological process of HCC through these tumor-associated pathways, providing a basis for molecular-level studies.

## Discussion

In this study, we identified 10 miRNAs associated with HCC recurrence and prognosis. The accuracy of these 10 miRNAs to predict tumor recurrence after surgery or liver transplantation in patients with HCC was verified using an external validation dataset (GSE30297) and an internal validation dataset (TCGA). Multiple previous studies have shown that these 10 miRNAs are associated with multiple malignancies, including hepatocellular carcinoma, gastric cancer, and pancreatic ductal adenocarcinoma [12–14].

These 10 miRNAs can accurately predict recurrence of hepatocellular carcinoma and, in addition, are associated with several tumor biological processes. Several miRNAs (such as hsa-miR-509-3p, hsa-miR-769-3p, and hsa-miR-342-3p) play a role in the inhibition of the proliferation, migration, invasion of breast cancer, glioma, and non-small cell lung cancer [15–17]. Multiple pathways are involved in the mechanism by which recurrence-associated miRNAs regulate malignancy progression, such as the tumor-associated pathways Wnt/β-catenin signaling pathway (hsa-miR-769-3p), ERK signaling (hsa-miR-193a-3p), and AKT-mTOR pathway (hsa-miR-193a-5p) [16, 18, 19]. These 10 miRNAs are also associated with drug resistance to antitumor drugs. For example, hsa-miR-509-3p regulates platinum drug sensitivity in ovarian cancer and osteosarcoma [20, 21]. A previous study suggested that hsa-miR-193a-5p plays a role in reducing 5-FU and oxaliplatin chemoresistance in colorectal cancer [22]. Our KEGG functional analysis results are consistent with previous studies showing that these miRNA-associated mRNAs are enriched in malignancy-related pathways such as hepatocellular carcinoma (hsa05225), pancreatic cancer (hsa05212), non-small cell lung cancer (hsa05223), and colorectal cancer (hsa05210).

For individual miRNAs, multiple previous studies have described their role in the recurrence and prognostic assessment of HCC. Levels of the oncogene hsa-miR-767-5p are significantly elevated in HCC tissues, and this high expression trend correlates with poor DFI and OS [23]. According to the results of our study, the expression level of hsa-miR-767-5p correlated negatively with prognosis. Another prognosis-related gene, hsa-miR-421, which is known as a tumor-promoting factor, was shown to be associated with worse prognosis in our study and a previous one [24]. However, there was a different trend for hsa-miR-296-5p. One study showed that higher expression of hsa-miR-296-5p is associated with a better prognosis, whereas our results indicated that high expression of hsa-miR-296-5p is associated with favorable DFI (*P* = 0.0027) but not with OS (*P* > 0.05) [25]. Similarly, there is inconsistency between a previous study and our findings for hsa-miR-342-3p [26]. More surprisingly, the findings of Kaplan‒Meier survival analysis of DFI and OS were opposite in our study. The most obvious reason for these differences is that miRNAs play a pleiotropic role in cancer. The intricacy of the genetic network makes it probable that several important miRNAs, as opposed to just one, are required for tumor growth. Therefore, the stability and reproducibility of integrating multiple miRNAs for recurrence and prognosis assessment of HCC patients are better than with single miRNAs.

Because postoperative recurrence is an important factor affecting the survival of HCC patients, researchers have been attempting to find miRNA combinations to accurately predict postoperative recurrence. Bai et al. identified a 6-miRNA signature (including hsa-miR-210, hsa-miR-550a-1, hsa-miR-3199–2, hsa-miR-4732, hsa-miR-22, and hsa-miR-139) to predict postoperative recurrence in patients with HCC [11]. Not only postoperative recurrence but also assessment of the prognosis of patients with HCC can guide clinicians in decisions. Based on imaging of vascular invasion and metastasis-related miRNAs, investigators have determined that different miRNA combinations well predict the prognosis of liver cancer patients [10, 27]. These two studies coincide with our idea in terms of variable inclusion selection. The miRNAs they included were metastasis or vascular invasion-associated miRNAs and not miRNAs differentially expressed in tumor tissue and normal tissue. We also believe that screening variables for recurrence and prognosis assessment should focus on the full range of miRNAs, rather than on differential miRNA expression between tumor tissue and normal tissue. The differential expression profile of miRNAs between tumor tissue and normal tissue should be the focus of research on malignant tumor diagnosis [28].

One of the main concerns reflecting the greater mortality of HCC is a recurrence after liver transplantation. A previous study has shown that circulating exosomal miR-92b is a good predictor of HCC recurrence after liver transplantation [29]. For hepatitis B virus-mediated hepatocellular carcinoma, miR-21 is associated with post-translation tumor recurrence [30]. We verified the role of these 10 miRNAs in the prediction of HCC recurrence after liver transplantation in the validation dataset. This is a kind of double validation, that is, it not only confirms the predictive ability of postoperative recurrence of HCC, but also verifies the predictive ability of relapse after liver transplantation.

The focus of most studies on tumor diagnosis and prognosis has always been the selection process of variables. Excellent variable screening methods can help researchers remove noise in the data matrix and avoid overfitting of the model. In this study, Lasso regression and Gaussian mixed model were used for variable screening, and the results showed that the method we chose was well suited for our study. In addition to our method of choice, bioinformatics engineers are also looking for disease-relevant specific miRNA screening methods. Computational prediction models might efficiently explore the most likely candidates for additional validation trials as a useful supplement to experiment-based techniques, which are expensive and time-consuming, and so reduce the time and cost for miRNA-disease association detection. These prediction models may specifically be broken down into four groups: models based on score functions, complicated network algorithms, machine learning, and numerous biological information sources [31]. Our research did not compare the pros and cons of these methods with our innovative Lasso regression analysis combined with Gaussian mixed model variable screening method, which had certain limitations. In further research we will focus on these computational prediction models to screen out disease-specific miRNAs.

When clinicians are faced with brand-new sequencing data and want to screen out disease-related miRNAs, the first thought is to find differentially expressed miRNAs, and then functional enrichment analysis to find specific pathways or function-related miRNAs, and then proceed to the next experiment. The possible reasons for this phenomenon mainly include that clinicians are unfamiliar with the underlying algorithms and lack the ability to skillfully use advanced statistics. This study also has this limitation. In the variable screening step, in order to avoid the exclusion of too many miRNAs in differential analysis and functional enrichment analysis, which may cause some miRNAs to be mistakenly considered as noises not related to disease, we included all miRNAs in the variable screening process. In recent years, computer scientists and algorithm engineers have developed many significant algorithmic models for predicting disease-related miRNAs. This provides a new idea for clinicians to screen disease-related miRNAs. For instance, a unique computational approach called Ensemble of Decision Tree based MiRNA-Disease Association prediction (EDTMDA) develops a computational framework for ensemble learning and dimensionality reduction, which can precisely screen certain disease-related miRNAs [32]. The computer approach of integrating heterogeneous biological data to anticipate probable correlations can significantly reduce time and money when compared to traditional biological tests. Deep-belief network for miRNA-disease association prediction model (DBNMDA) was developed by researchers. It used feature vectors to pre-train restricted Boltzmann machines for all miRNA-disease pairs and applied positive samples and the same number of selected negative samples to fine-tune DBN to produce the final predicted scores [33]. In addition, a brand-new computational model called Neighborhood Constraint Matrix Completion for MiRNA-Disease Association prediction (NCMCMDA) has been developed, whose basic theory is to recover the missing miRNA-disease associations based on the known miRNA-disease associations and integrated disease (miRNA) similarity [34]. These disease-related miRNA screening methods based on complex algorithms and excellent prediction accuracy provide clinicians with new options.

The prognosis of HCC, or the use of bioinformatics methods to build a predictive model for predicting the overall survival or disease-free survival of patients with HCC, is currently the main focus of research on the screening of HCC-related diagnostic molecules based on public databases. Additionally, the Lasso regression analysis is the primary variable screening technique. In this study, the prediction of HCC recurrence-which is novel in other studies-includes the prediction of HCC recurrence following liver transplantation. Lasso regression analysis and a Gaussian mixed model were innovatively used in this study's variable screening, and the validation findings demonstrate that the model created using this variable screening approach also performed well in the validation dataset. However, there are certain drawbacks to our study, the most significant of which is the lack of our own validation data in the internal and validation datasets, which were collected from public databases. In order to build more advanced mathematical models for the diagnosis, prognosis, and recurrence of HCC patients, we will gather the clinical data of patients with HCC at our facility and create our own sequencing database in the course of future research.

## Conclusion

In conclusion, the combination of 10 miRNAs can accurately predict tumor recurrence in HCC patients after surgery and liver transplantation. Moreover, these 10 miRNAs also play a guiding role in the prognosis evaluation of HCC patients. Further studies are required to validate our recurrence and prognosis assessment model using a large-scale validation dataset, and to confirm the biological functions of these 10 miRNAs through in vivo and in vitro experiments.

## Data Availability

The datasets used in this study were downloaded from GEO (https://www.ncbi.nlm.nih.gov/geo/) and UCSC xena (http://xena.ucsc.edu/). The R package used was obtained from Bioconductor (https://www.bioconductor.org/). All data generated or analyzed during this study are included in this published article. If readers have any questions about the data processing, please do not hesitate to contact us (Xuanfeng Zhang: zxfujs@126.com).
